# Recommended next care following hospital-treated self-harm: Patterns and trends over time

**DOI:** 10.1371/journal.pone.0193587

**Published:** 2018-03-01

**Authors:** Ella Arensman, Eve Griffin, Caroline Daly, Paul Corcoran, Eugene Cassidy, Ivan J. Perry

**Affiliations:** 1 National Suicide Research Foundation, Cork, Ireland; 2 School of Public Health, University College Cork, Cork, Ireland; 3 Department of Psychiatry, University College Cork, Cork, Ireland; 4 Liaison Psychiatry Service, Cork University Hospital, Cork, Ireland; University of Queensland, AUSTRALIA

## Abstract

**Objective:**

The specific objectives of this study were to examine variation in the care of self-harm patients in hospital settings and to identify the factors that predict recommended next care following self-harm.

**Methods:**

Data on consecutive presentations to Irish emergency departments (EDs) involving self-harm from the National Self-Harm Registry Ireland from 2004 to 2012 were utilised. Univariate and multivariate regression analyses were performed to assess the associations between patients’ clinical and demographic characteristics, and recommended next care received.

**Results:**

Across the study period a total 101,904 self-harm presentations were made to hospital EDs, involving 63,457 individuals. Over the course of the study there was a declining number of presentations resulting in patient admission following attendance with self-harm. Recommended next care varied according to hospital location, with general admission rates ranging from 11% to 61% across administrative health regions. Multinomial logistic regression identified that the factor which most strongly affected next care was the presenting hospital. Being male, older age, method, repeat self-harm, time of attendance and residence of the patient were all identified as influencing care received. Psychiatric admission was most common when highly lethal methods of self-harm were used (OR = 4.00, 95% CI, 3.63–4.41). A relatively large proportion of patients left the ED without being seen (15%) and the risk of doing so was highest for self-harm repeaters (1.64, 1.55–1.74 for those with 5+ presentations).

**Conclusions:**

The extensive hospital variation in recommended next care indicates that management of self-harm patients may be determined more by where they present than by the needs of the patient. The study outcomes underline the need to standardise the clinical management of self-harm patients in general hospital settings.

## Introduction

Presentations of self-harm represent a substantial number of all hospital emergency department (ED) visits.[1–3] International guidelines for the short-term management of self-harm in emergency settings support and advocate the standardised assessment and management of self-harm.[4,5] However, previous studies have found regional variation in the admission rates and assessment procedures following hospital-treated self-harm, with rates of psychosocial assessment ranging from 36–82%.[6–8] Such variation suggests that care frequently appears to be below the standard recommended.[9–11] Clinical management and recommended next care of self-harm has also been shown to vary both by patient characteristics such as method of self-harm,[12] as well as by the presenting hospital.[13] Furthermore, there is limited evidence to suggest that clinical management of self-harm is associated with improved outcomes for self-harm patients.[14]

The focus of previous studies on this topic have tended to be narrow—for example, focusing on hospital admission as a sole outcome [15–17] or specific methods of self-harm.[12] Longitudinal data obtained by the Irish National Self-Harm Registry provides the opportunity to examine patterns of recommended next care for hospital-treated self-harm. The specific study objectives were to examine variation in the management of self-harm patients based on standard demographic and clinical characteristics; to map regional variation in recommended next following self-harm at national level and to examine trends over time; and to identify the factors that predict recommended next care.

## Method

### National Self-Harm Registry Ireland

Data for the period 2004 to 2012 were obtained from the National Self-Harm Registry Ireland. Between 2004 and 2005 the Registry had near-complete coverage of the hospital EDs in Ireland, with all hospitals contributing data to the Registry since 2006. A weighting was applied to adjust for the lack of data from two hospitals in 2004 and 2005.

### Definition of self-harm

The Registry uses the following definition of self-harm: “an act with non-fatal outcome in which an individual deliberately initiates a non-habitual behaviour, that without intervention from others will cause self harm, or deliberately ingests a substance in excess of the prescribed or generally recognised dosage, and which is aimed at realising changes that a person desires via the actual or expected physical consequences”.[18] This definition was developed by the WHO/Euro Multicentre Study and is consistent with that used in the English multicentre study.[2] A repeat episode is defined as re-presentation to any ED due to self-harm after an index episode of self-harm.

Self-harm data is collected and collated by independent Data Registration Officers according to standardised operating procedures.^3^

### Data items

The Registry has a core dataset including: gender, age, date and hour of attendance at hospital and method(s) of self-harm according to the tenth revision of the WHO’s International Classification of Disease codes for intentional injury (X60-X84). In addition, patient initials (in an encrypted format) and area of residence, coded to administrative area, are recorded.

Recommended next care following treatment to the presenting ED is routinely recorded by the Registry. The outcomes that were captured included: a) admission to a general ward in the presenting hospital; b) admission to a psychiatric ward in the presenting hospital; c) patient refusing admission/ leaving without being seen; and d) discharge from ED following treatment.

Whether or not alcohol was consumed as part of the self-harm act was ascertained through hospital case notes—if it was recorded on registration by the attending clinician or if present on toxicology reports.

### Data analysis

Univariate analyses were performed using chi-square tests with a significance level set at p<0·01. A multinomial logistic regression model was developed to assess the associations between patients’ clinical and demographic characteristics, and recommended next care received in order to identify which factors differentiated between general admission, psychiatric admission, leaving without being seen/refusing admission, and discharged from the ED. The dependent variable comparison group represented those patients discharged from the ED. Odds Ratios and their 95% Confidence Intervals were reported with the associated level of significance. All statistics were performed using SPSS Version 21.

### Mapping of regional variations in recommended next care

**Fig 1:**
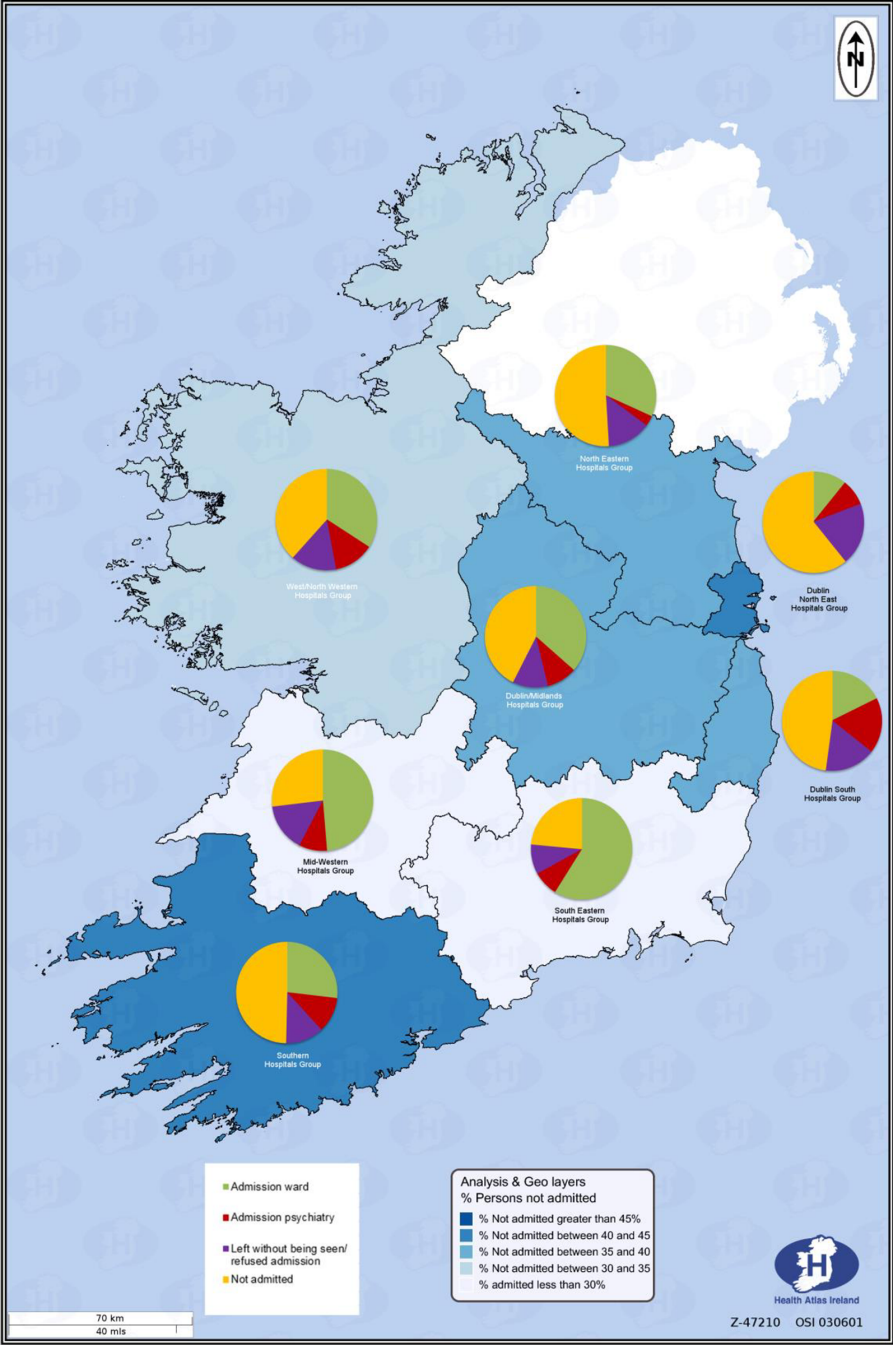
Regional variation of recommended next care of hospital-treated self-harm by HSE Hospital Group, average 2004–2012.

The map (Fig 1) details the recommended next care following hospital-treated self-harm, according to the Health Service Executive (HSE) Hospital Groups in Ireland. The map was developed using Health Atlas (www.healthatlasireland.ie).

### Ethical approval

Ethical approval has been granted by the National Research Ethics Committee of the Faculty of Public Health Medicine, Ireland and relevant hospital and regional ethics committees. The National Suicide Research Foundation is registered with the Irish Data Protection Agency and complies with the Irish Data Protection Act of 1988 and the Irish Data Protection (Amendment) Act of 2003. Only anonymised data are released in aggregate form in reports. The names and addresses of patients are not recorded.

## Results

During the nine-year period 1^st^ January 2004 to 31^st^ December 2012, there were 101,904 self-harm presentations to hospital recorded by the Registry, involving 63,457 individuals. Intentional drug overdose was the most common method of self-harm, present in 72% of all episodes. Self-cutting was the only other common method, involved in 22% of all presentations.

Table 1 shows the patterns of recommended next care following self-harm, based on demographic, temporal and clinical characteristics. Most commonly, 41% of presentations were discharged from the ED following treatment. A similar proportion (44%) resulted in admission to either a general ward (33%) or a psychiatric unit (11%). In 15% of presentations, the patient either refused to be admitted or left the ED before a next care recommendation could be made. Patterns of recommended next care varied significantly across the study period [*X*^*2*^ for trend (1) = 46.14; p<0.001], with general ward admission decreasing by 30% (from 40% to 28%) (see also S1 Fig).

**Table 1:** Demographic and clinical characteristics according to recommended next care received.

	Discharge from ED (n = 42,047; 41.3%)		General admission (n = 33,348; 32.7%)		Psychiatric admission (n = 11,592; 11.4%)		Left without being seen/ refuse admission (n = 14,917; 14.6%)		X^2^(df)
	n	(%)	n	(%)	n	(%)	n	(%)	
**Gender**									
Male	19010	(41.0%)	14103	(30.4%)	5616	(12.1%)	7637	(16.5%)	
Female	23037	(41.5%)	19245	(34.7%)	5976	(10.8%)	7280	(13.1%)	375·77(3), P<0.001
**Age (years)**									
<15	759	(37.5%)	1132	(55.9%)	54	(2.7%)	79	(3.9%)	
15–24	14845	(47.8%)	9283	(29.9%)	2696	(8.7%)	4247	(13.7%)	
25–34	10618	(41.4%)	7623	(29.7%)	3252	(12.7%)	4132	(16.1%)	
35–44	8263	(37.8%)	7133	(32.6%)	2785	(12.7%)	3666	(16.8%)	
45–54	4993	(36.0%)	5015	(36.2%)	1765	(12.7%)	2098	(15.1%)	
55+	2569	(34.4%)	3162	(42.4%)	1040	(13.9%)	695	(9.3%)	367·81(1), P<0.001
**Presentation**									
1^st^	26929	(42.4%)	22114	(34.8%)	6103	(9.6%)	8311	(13.1%)	
2^nd^	5780	(39.2%)	4942	(33.5%)	1874	(12.7%)	2159	(14.6%)	
3^rd^	2549	(38.6%)	2037	(30.9%)	956	(14.5%)	1054	(16.0%)	
4^th^	1459	(38.5%)	1121	(29.6%)	567	(15.0%)	640	(16.9%)	
5^th^ or subsequent	5330	(40.0%)	3134	(23.5%)	2092	(15.7%)	2753	(20.7%)	186·12(1), P<0.001
**Method**									
Drug overdose	25028	(37.4%)	26855	(40.1%)	5613	(8.4%)	9402	(14.1%)	
Self-cutting	9608	(56.8%)	200	(11.8%)	2321	(13.7%)	2978	(17.6%)	
Drug overdose and self-cutting	1854	(42.2%)	1287	(29.3%)	556	(12.6%)	700	(15.9%)	
Attempted hanging	1282	(39.0%)	639	(19.4%)	995	(30.3%)	370	(11.3%)	
Attempted drowning	833	(38.8%)	407	(18.9%)	550	(25.6%)	359	(16.7%)	
Other	3442	(41.6%)	2160	(26.1%)	1557	(18.8%)	1108	(13.4%)	7644·56(15), P<0.001
**Alcohol involvement**									
Yes	16121	(39.1%)	14426	(35.0%)	3578	(8.7%)	7131	(17.3%)	
No	25926	(42.7%)	18922	(31.2%)	8014	(13.2%)	7786	(12.8%)	963·57(3), P<0.001
**Residence**									
Household resident	38437	(41.2%)	31356	(33.6%)	9993	(10.7%)	9993	(14.4%)	
Hospital in-patient	205	(16.5%)	268	(21.6%)	732	(58.9%)	38	(3.1%)	
Prisoner	233	(68.7%)	76	(22.4%)	6	(1.8%)	6	(7.1%)	
Homeless	1360	(44.7%)	490	(16.1%)	396	(13.0%)	396	(26.2%)	
Other	847	(45.4%)	515	(27.6%)	217	(11.6%)	217	(15.3%)	3562·93(12), P<0.001
**Year of presentation**									
2004	3117	(30.4%)	4134	(40.3%)	1497	(14.6%)	1521	(14.8%)	
2008	5131	(44.0%)	3836	(32.9%)	1185	(10.2%)	1517	(13.0%)	
2012	5723	(47.7%)	3311	(27.6%)	1241	(10.3%)	1735	(14.4%)	46·14(1), P<0.001
**Time of presentation**									
12am-3am	9719	(42.1%)	7254	(31.5%)	2075	(9.0%)	4017	(17.4%)	
4am-7am	4652	(42.9%)	3588	(33.1%)	986	(9.1%)	1621	(14.9%)	
8am-11am	3757	(43.5%)	3028	(35.1%)	1086	(12.6%)	757	(8.8%)	
12pm-3pm	6561	(42.3%)	4944	(31.9%)	2191	(14.1%)	1800	(11.6%)	
4pm-7pm	7686	(39.2%)	6492	(33.1%)	2560	(13.1%)	2851	(14.6%)	
8pm-11pm	9453	(40.2%)	7658	(32.5%)	2599	(11.0%)	3819	(16.2%)	19·17(1), P<0.001
**Weekend presentation**									
Yes	12909	(41.4%)	10216	(32.7%)	3444	(11.0%)	4634	(14.9%)	
No	29138	(41.2%)	23132	(32.7%)	8148	(11.5%)	10283	(14.5%)	6·04(3), P = .110
**City resident**									
Yes	14404	(46.9%)	6202	(20.2%)	3822	(12.4%)	6316	(20.5%)	
No	27643	(38.8%)	27146	(38.1%)	7770	(10.9%)	8601	(12.1%)	3545·14(3), P<0.001

Note: Presentation variable indicates whether it was a patient’s first, second or subsequent attendance with self-harm. ‘Other’ residence would include non-residents, such as tourists and visitors. Data presented for year of presentation includes first, middle and last years of study period only.

Recommended next care varied according to gender (*X*^*2*^(3) = 375.77; p<0.001). Overall, females were more likely to be admitted to a general ward than males (34.7% vs. 30.4%). Males more often left the ED before a next care recommendation could be made or they refused treatment (16.5% vs. 13.1%). Recommended next care also varied significantly by age [*X*^*2*^ for trend (1) = 367.81; p<0.001], with the rate of general admission to a hospital ward being highest for children and adolescents under 15 years (56%) and for those aged over 55 years (42%). Almost half of those aged between 15–24 years were discharged from the ED following treatment. In addition, psychiatric admission following a self-harm presentation was lowest among those aged under 15 years (2.7%).

Next care also varied according to method of self-harm [*X*^*2*^(15) = 7644.56, p<0.001]. General inpatient admission was most common following cases of poisoning, drug overdose and in cases where alcohol was involved (40%, 39% and 35%, respectively), and least common following self-cutting (16%). Psychiatric admission was most common following cases of attempted hanging and attempted drowning (30% and 25%, respectively). Patients were most likely to leave the ED without being seen if alcohol was involved (17.3% vs. 12.8%) or if the episode of self-harm involved self-cutting only (16.7%).

Admission was significantly associated with time of presentation, with variation in both general (32.5% to 39.8%) and psychiatric (9.0% to 14.1%) admission rates over the course of the day, and in the proportion of patients leaving without being seen/ refusing admission (8.8% to 17.4%).

### Regional variation of recommended next care

Recommended next care varied by the Health Service Executive Hospital Groups where the presenting hospital was located (Fig 1). General admission ranged from 11.2% in the Dublin North East Hospital Group to 61.0% in the South Eastern Hospital Group. Admission to a psychiatric ward was lowest (3.7%) in the North Eastern Hospital Group and highest in the Dublin South Hospital Group (19.3%). The Dublin North East Hospital Group had the highest proportion of patients leaving the ED without being seen or without a clinical decision being made (18.9%).

### Factors associated with next care following hospital-treated self-harm

Table 2 details the results of the multivariate multinomial regression which was conducted to identify the factors independently associated with self-harm patients and different types of recommended next care. The analysis controlled for the hospital where the presentation was made, and all 38 hospitals were included in the regression model. This variable was most strongly associated with recommended next care, with Odds Ratios (ORs) ranging from 0.04 to 2.38 (see S2–S4 Figs).

**Table 2:** Multinomial logistic regression analysis of the independent associations of factors and recommended next care following hospital-treated self-harm.

Variable	General admission		Psychiatric admission		Left without being seen/ refuse admission	
	OR	(95% CI)	OR	(95% CI)	OR	(95% CI)
**Gender (female)**						
**Male**	1·10*	(1·06–1·13)	1·15*	(1·10–1·21)	1·30*	(1·25–1·35)
**Age in years (55+)**						
**<15**	1·11	(·97–1·27)	0·18*	(0·13–0·26)	0·48*	(0·36–0·64)
**15–24**	0·45*	(0·42–0·49)	0·43*	(0·39–0·47)	1·01	(0·92–1·11)
**25–34**	0·57*	(0·53–0·61)	0·71*	(0·65–0·78)	1·28*	(1·16–1·40)
**35–44**	0·66*	(0·61–0·70)	0·80*	(0·73–0·87)	1·39*	(1·26–1·52)
**45–54**	0·80*	(0·74–0·86)	0·88*	(0·80–0·97)	1·38*	(1·25–1·53)
**Presentation (1**^**st**^**)**						
**2**^**nd**^	1·13*	(1·07–1·19)	1·44*	(1·35–1·54)	1·20*	(1·31–1·27)
**3**^**rd**^	1·12*	(1·05–1·21)	1·63*	(1·50–1·78)	1·32*	(1·22–1·43)
**4**^**th**^	1·11	(1·01–1·22)	1·67*	(1·50–1·87)	1·38*	(1·25–1·53)
**5**^**th**^ **or subsequent**	0·96	(0·91–1·02)	1·64*	(1·53–1·75)	1·64*	(1·55–1·74)
**Method (drug overdose)**						
**Self-cutting**	0·16*	(0·15–0·17)	0·99	(0·93–1·05)	0·77*	(0·73–0·81)
**Drug overdose and self-cutting**	0·70*	(0·64–0·76)	1·45*	(1·30–1·61)	0·92	(0·83–1·01)
**Attempted hanging**	0·45*	(0·40–0·50)	4·00*	(3·63–4·41)	0·75*	(0·66–0·85)
**Attempted drowning**	0·36*	(0·32–0·41)	2·95*	(2·61–3·33)	0·93	(0·81–1·06)
**Other**	0·57*	(0·54–0·61)	2·04*	(1·90–2·20)	0·82*	(0·76–0·88)
**Alcohol involvement (No)**						
**Yes**	0·95	(0·92–0·99)	0·68*	(0·65–0·71)	1·24*	(1·19–1·29)
**Residence (Household resident)**						
**Hospital in-patient**	1·75*	(1·42–2·15)	9·61*	(8·11–11·38)	0·47*	(0·33–0·66)
**Prisoner**	0·39*	(0·29–0·52)	0·05*	(0·22–0·12)	0·25*	(0·16–0·39)
**Homeless**	0·67*	(0·60–0·76)	0·80*	(0·71–0·90)	1·14	(1·04–1·26)
**Other**	0·69*	(0·60–0·78)	0·65*	(0·55–0·76)	0·89	(0·77–1·02)
**Year of presentation (2004)**						
**2008**	0·55*	(0·52–0·60)	0·41*	(0·37–0·45)	0·55*	(0·50–0·60)
**2012**	0·44*	(0·41–0·47)	0·38*	(0·34–0·41)	0·55*	(0·51–0·60)
**Time of presentation (8pm-11pm)**						
**12am-3am**	0·95	(0·90–0·99)	0·85*	(0·79–0·91)	1·00	(0·95–1·06)
**4am-7am**	0·97	(0·92–1·04)	0·87	(0·80–0·95)	0·85*	(0·79–0·91)
**8am-11am**	1·10	(1·03–1·17)	1·11	(1·02–1·21)	0·54*	(0·50–0·59)
**12pm-3pm**	0·94	(0·89–1·00)	1·20*	(1·11–1·28)	0·71*	(0·67–0·76)
**4pm-7pm**	1·03	(0·98–1·09)	1·18*	(1·11–1·27)	0·94	(0·89–1·00)
**Weekend presentation (Yes)**						
**No**	0·97	(0·94–1·01)	1·00	(0·95–1·05)	0·98	(0·93–1·02)
**City resident (No)**						
**Yes**	0·90	(0·85–0·94)	1·00	(0·94–1·07)	1·28*	(1·21–1·35)

* = p<0·001. Note: Reference category for dependent variable is ‘discharge from ED’. Reference categories for independent variables shown in brackets. All factors listed were entered into the multivariate model.

Gender was independently associated with recommended next care, with males more likely to be admitted to both general (OR = 1.10, 95% CI, 1.06–1.13) and psychiatric wards (1.15, 1.10 to 1.21) than females. In addition, males were more likely to leave the ED without being seen or refuse admission (1.30, 1.25–1.35). The likelihood of being admitted to a psychiatric ward increased with increasing age, and was lowest for those aged under 15 years (0.18, 0.13–0.26). A similar trend was observed for general admission (0.45, 0.42–0.49 for 15–24 year-olds), however those aged under 15 years were just as likely as older adults (55+) to be admitted to a general ward. Young and middle-aged adults (25–54 years) were most likely to leave the ED without being seen or to refuse admission, with those aged under 15 least likely to do so (0.48, 0.36–0.64). Patients residing in psychiatric hospitals were most likely to be both admitted to general (1.75, 1.42–2.15) and psychiatric (9.61, 8.11–11.38) wards. City residents were more likely to leave the ED without being seen (1.28, 1.21–1.35).

General and psychiatric admission following self-harm decreased over the study period, as did the proportion of those leaving without being seen/refusing admission. Presentations made between 12pm and 7pm (12pm-3pm: 1.20, 1.11–1.28; 4pm-7pm: 1.18, 1.11–1.27, respectively) were most likely to receive psychiatric admission, while those attending between 4am and 3pm were least likely to leave without being seen by a doctor. Whether the attendance occurred during the week or at the weekend did not significantly influence next care recommendations.

Characteristics of the self-harm presentation also influenced recommended next care. A person was most likely to be admitted to a general ward following intentional drug overdose, and least likely if self-cutting was involved (0.16, 0.15–0.17). Attempted hanging was most strongly associated with psychiatric admission (4.00,3.63–4.41), while those engaging in self-cutting were least likely to leave without being seen (0.77, 0.73–0.81). Having used/misused alcohol was associated with leaving without being seen (1.24, 1.19–1.29). The number of previous presentations a person had made also influenced next care. The likelihood of being admitted to a psychiatric ward increased with each subsequent attendance for self-harm (5^th^ or subsequent attendance: 1.64, 1.53–1.75). For admission to a general ward, only those patients on their second or third attendance were more likely to be admitted (1.13, 1.07–1.19; 1.12, 1.05–1.21, respectively). In contrast, patients on a repeat presentation were also more likely to leave the ED without being seen or to refuse admission (5^th^ or subsequent attendance: 1.64, 1.55–1.74).

## Discussion

This is the first study addressing recommended aftercare following self-harm presenting to general hospital at national level. The findings of this study indicate that over time there was a decreasing number of presentations resulting in inpatient admission to the presenting hospital following self-harm. In addition, significant variation in the patterns of recommended next care was observed across hospitals. The factor most strongly associated with recommended next care was the hospital to which the individual presented. The study also identified that being male, older age, method of self-harm, repeat attendance, time of attendance and residence of the patient all influenced the type of care patients received. Males were more likely to be admitted to hospital, and also more likely to leave without being seen, than females. Older patients were more likely to be admitted to hospital following self-harm, with those aged between 25–44 years most likely to leave without being seen. Psychiatric admission following self-harm was most common when the presentation was made during daytime hours.

This study identified a sub-group of patients who were at greatest risk of leaving the ED without being seen, or refusing admission. These patients were most likely to be male and aged between 25–44 years. These presentations were more likely to involve an intentional drug overdose or attempted drowning, often involving alcohol. City residents, those with no fixed abode, and those with a history of previous self-harm, were more likely to leave without treatment. Patients presenting due to self-harm in the evening or at night time were also more likely to leave without treatment.

Our study has a number of strengths and limitations. Firstly, Ireland’s National Self-Harm Registry is the world’s first national registry focusing specifically on non-fatal self-harm presenting to hospital EDs and provides a unique opportunity to establish, at a national level, the patterns of recommended next care in hospitals involving non-fatal suicidal behavior.[19] Secondly, over 100,000 presentations of self-harm made over a nine-year period were included in this analysis, providing robust data to examine longitudinal patterns of care for self-harm patients. A limitation of the study is the lack of information on psychosocial assessments offered to patients during their ED presentation, as well as on referrals provided to patients who were discharged from the presenting hospital. This information was not included in the data collection for the Registry during the study period. More detailed clinical data relating to psychiatric history, degree of suicidal intent, severity of the self-harm act and contributory factors were not recorded. To this end, we were not able to quantify their impact on recommended next care and acknowledge that these would be important to consider. Additionally, it may not always be recorded in the ED that a patient has been admitted directly to psychiatric inpatient care, or a patient may be admitted to an external psychiatric hospital for admission. Therefore, the figures presented for psychiatric admissions could be underestimated. Finally, establishing self-harm history was confined to presentations made that were recorded by the Registry database, thus assuming that a person’s first episode in the Registry was their first presentation to hospital for self-harm.

To our knowledge only one other recent study has examined patterns of recommended next care for self-harm using a multinomial approach.[16] However, our research is based on national data and covers a longer time period. The variation in recommended next care across hospitals was similar to other studies.[6–8] However, general ward admissions were higher than those observed in a Spanish study [16] and lower than those reported in England,[6–8] and the improvement in admission rates in England since 2001 (42% to 54%)[8] was not reflected in our data. The independent risk factors for recommended next care following self-harm were consistent with previous literature.[17] In addition, the present study identified specific factors that were associated with increased likelihood of leaving the ED without being seen, which has not been examined previously. The observed variation in recommended next care across the study period and by hospital is undoubtedly complex and many contributing factors exist. The reduction in hospital admissions was most pronounced between 2008 and 2012, where numbers of general admission rates decreased by 16%. During this period of global economic recession, self-harm presentations increased by 31% for males and 22% for females.[20,21] The austerity measures introduced to Ireland during this time period involved substantial cuts in health care spending which may have impacted negatively on hospital resources, such as the availability of hospital beds and qualified staff to conduct psychosocial and psychiatric assessments. It is possible that the reduction in admissions during the study period was related to patients more often being treated in outpatient and community-based settings. We did not have access to data regarding referrals for discharged patients to examine this. However, given that the proportion of presentations resulting in the patient leaving the ED without being seen did not improve during the study period, it is unlikely that a reduction in admissions is fully attributable to service improvements.

The variation in recommended next care by hospital highlighted by the present study, poses a significant challenge for the assessment and management of self-harm in ED settings. The lowest admission rates following self-harm were observed in urbanised areas, coupled with a large proportion of patients leaving without being seen. In recent years, concerns have been expressed in the national media in relation to ED overcrowding in urban areas and to low per capita level of inpatient beds.[22] The recent Irish National Clinical Programme for the Management of Self Harm Presentations to Emergency Departments [23] aims to develop a standardised and effective process for the assessment and management of self-harm in an ED setting, and a specific aim of the programme to reduce the number of people who leave before assessment and reduce the number of repeat presentations. In terms of capacity building, new clinical posts have been allocated to each ED based on the data from the Registry to implement the programme and this resource will have a significant impact on availability of specialist staff on-site. A key to success of this programme will be effective implementation along with staff training as it is worth noting that in England, despite the comprehensive National Institute for Clinical Excellence (NICE) guidelines,[4] the proportion of patients who received a psychosocial assessment prior to leaving the ED did not significantly change.^8^

The present study highlights the variation in recommended next care for patients of self-harm according to previous self-harm history. Those who engaged in their second or third presentation were most likely to be admitted to a general ward, and those on their fourth or subsequent visit most likely to be admitted to a psychiatric ward. However, infrequent (less than five presentations) and frequent (five or more presentations) were all more likely to leave the ED without being seen, or to refuse admission, than those presenting on their index episode. This raises questions about the delivery and quality of initial assessments and current treatment for self-harm repeaters as well as the lack of awareness of the importance of self-harm and suicide risk assessment among these patients. Therefore, it is argued here that there is need for uniform assessment and referral procedures, in line with international best practice [4,5,24] to ensure the most appropriate treatment. For example, for patients with a pattern of frequent repetition of self-harm, Dialectical Behaviour Therapy is an indicated treatment.[25] However, if many of these patients do not receive an assessment at all, the likelihood of these patients receiving the required treatment is low.

In line with previous research, the hospital to which patients present appears to significantly influence the type of care and treatment a self-harm patient receives. In our study, data on hospital characteristics were lacking. Future research could use multi-level modelling approaches to explore specific hospital characteristics which affect the next care of self-harm. This research has identified that while repeaters of self-harm are more likely to be admitted to the presenting hospital, this occurs up to a point. Future research needs to explore the challenges associated with reaching this group of patients, and to ensure that the most appropriate treatment is being provided. There is varying evidence for the effectiveness of psychosocial assessments at the time of presentation in reducing repetition of self-harm.[14] Failure to routinely conduct a thorough and standardised psychosocial assessment of all self-harm patients may represent a missed opportunity to reduce the risk of both non-fatal and fatal repetition.[26] The Registry also has the potential to establish the effectiveness of out-patient referrals on repetition of self-harm, and future research should address this.

The results of this study show that non-clinical factors of self-harm presentations strongly influence patterns of hospital admission following self-harm. In particular, the hospital variation in recommended next care poses a challenge for the assessment and management of self-harm patients, and underlines the need for uniform guidelines.

## Supporting information

S1 Fig Changes in recommended next care of hospital-treated self-harm in Ireland, 2004–2012. (TIF)

S2 Fig Adjusted odds ratios by hospital for general admission. (TIF)

S3 Fig Adjusted odds ratios by hospital for psychiatric admission. (TIF)

S4 Fig Adjusted odds ratios for by hospital left without being seen/ refuse admission. (TIF)
