# Supplementary figures and images for: Recommended next care following hospital-treated self-harm: Patterns and trends over time
Source: PLoS One. 2018 Mar 1;13(3):e0193587. doi: 10.1371/journal.pone.0193587 (PMC5832269; doi:10.1371/journal.pone.0193587)

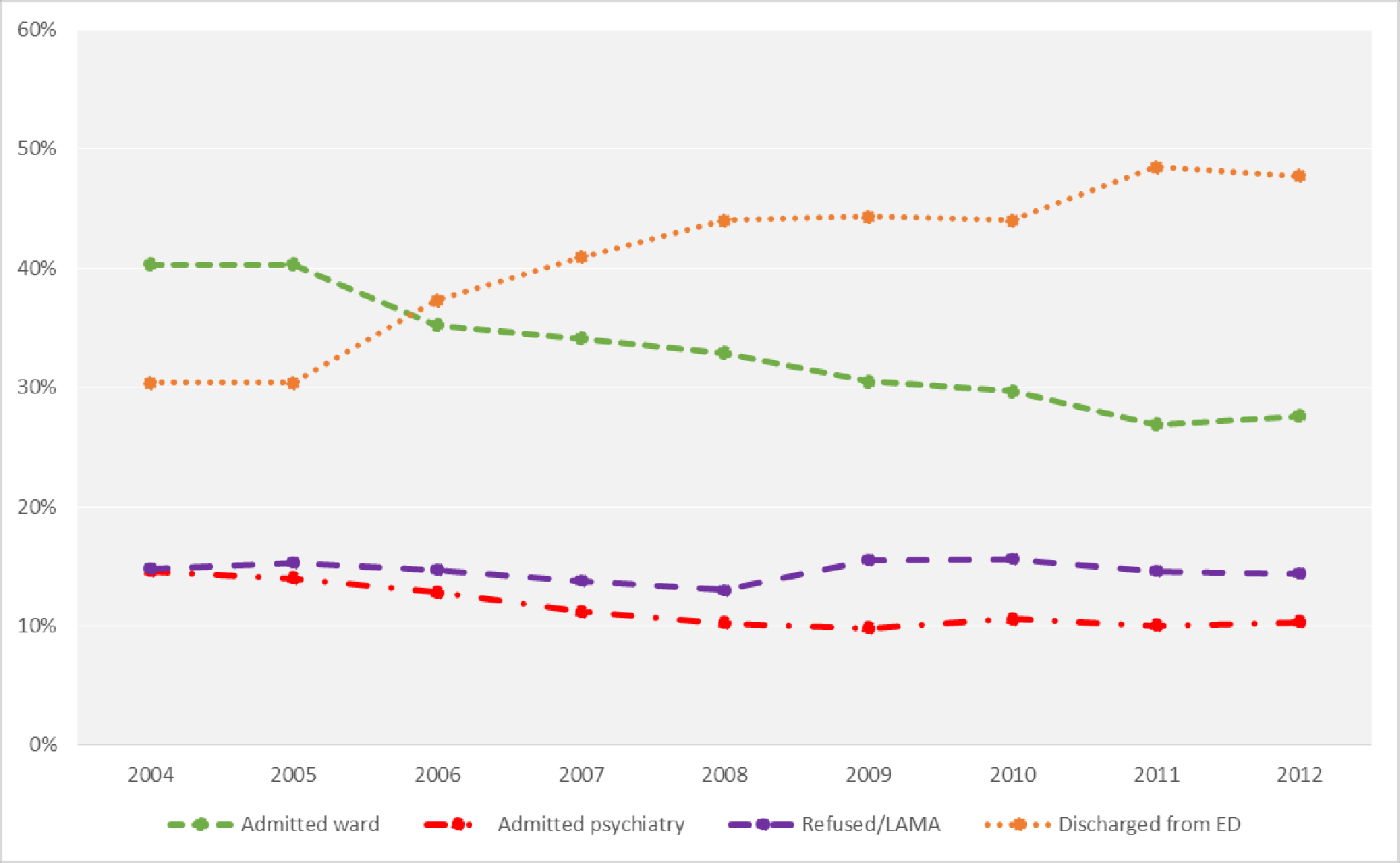

Supplement: S1 Fig — (TIF) [file pone.0193587.s001.tif]

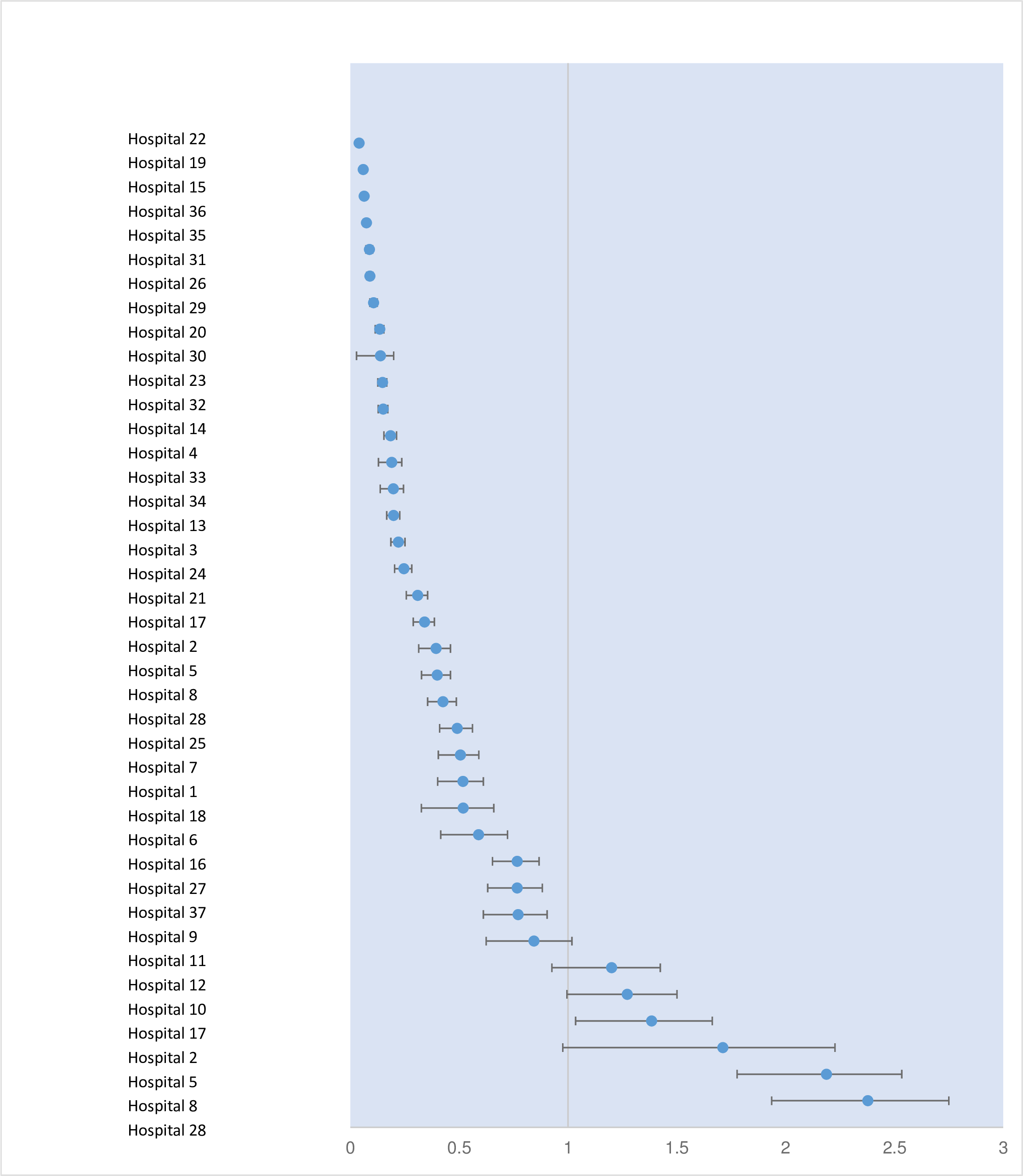

Supplement: S2 Fig — (TIF) [file pone.0193587.s002.tif]

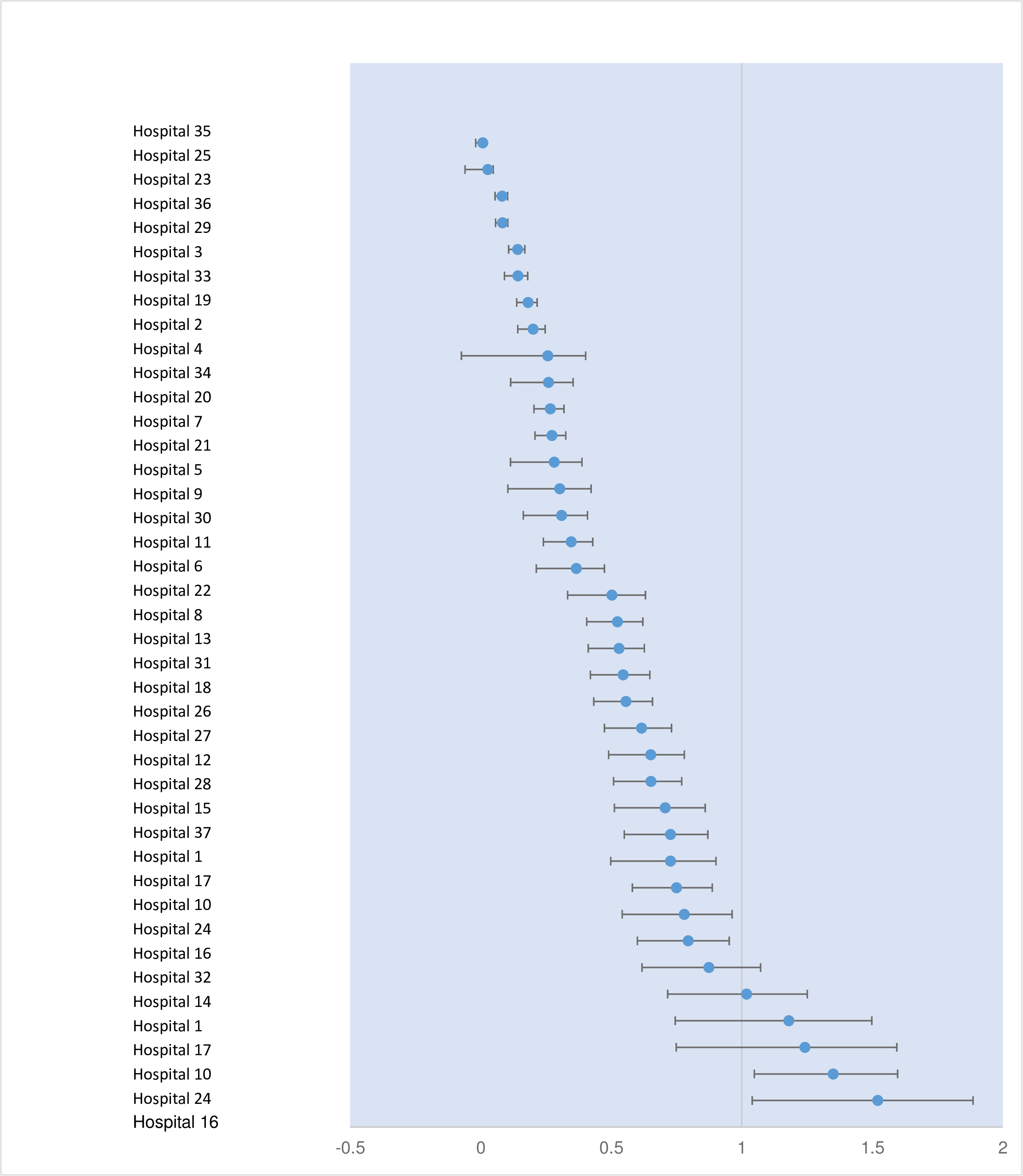

Supplement: S3 Fig — (TIF) [file pone.0193587.s003.tif]

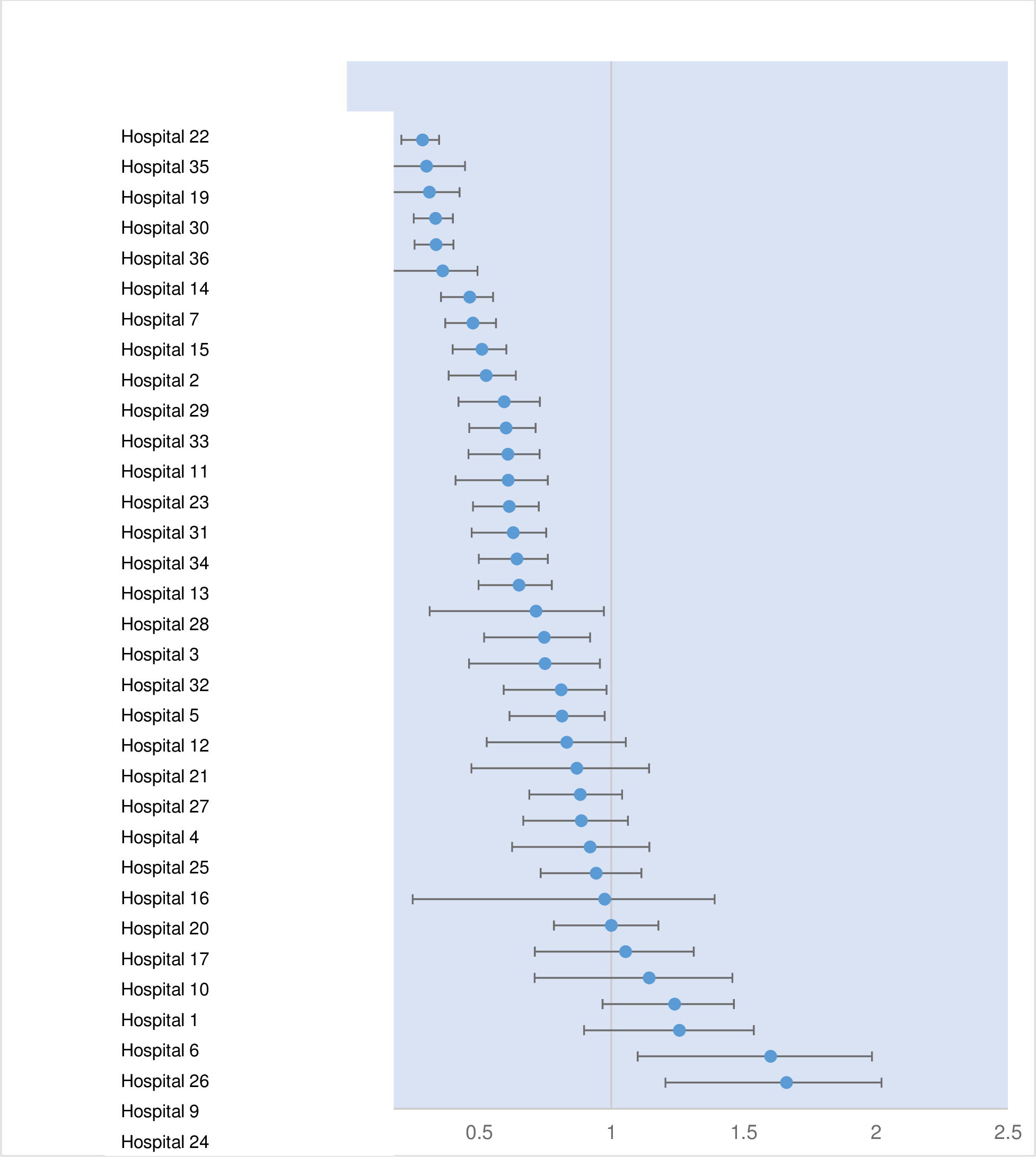

Supplement: S4 Fig — (TIF) [file pone.0193587.s004.tif]
